# Crowd Detection in Mass Gatherings Based on Social Media Data: A Case Study of the 2014 Shanghai New Year’s Eve Stampede

**DOI:** 10.3390/ijerph17228640

**Published:** 2020-11-20

**Authors:** Jiexiong Duan, Weixin Zhai, Chengqi Cheng

**Affiliations:** 1 School of Earth and Space Sciences, Institute of Remote Sensing and Geographical Information Systems, Peking University, Beijing 100871, China; duanjx@pku.edu.cn; 2 College of Information and Electrical Engineering, China Agricultural University, Beijing 100083, China; 3 Academy for Advanced Interdisciplinary Studies, Peking University, Beijing 100871, China; 4 College of Engineering, Peking University, Beijing 100871, China; ccq@pku.edu.cn

**Keywords:** geographic user-generated content, emergency, crowd aggregation, spatial analysis, sentiment analysis

## Abstract

The Shanghai New Year’s Eve stampede on 31 December 2014, caused 36 deaths and 47 other injuries, generating attention from around the world. This research aims to explore crowd aggregation from the perspective of Sina Weibo check-in data and evaluate the potential of crowd detection based on social media data. We develop a framework using Weibo check-in data in three dimensions: the aggregation level of check-in data, the topic changes in posts and the sentiment fluctuations of citizens. The results show that the numbers of check-ins in all of Shanghai on New Years’ Eve is twice that of other days and that Moran’s I reaches a peak on this date, implying a spatial autocorrelation mode. Additionally, the results of topic modeling indicate that 72.4% of the posts were related to the stampede, reflecting public attitudes and views on this incident from multiple angles. Moreover, sentiment analysis based on Weibo posts illustrates that the proportion of negative posts increased both when the stampede occurred (40.95%) and a few hours afterwards (44.33%). This study demonstrates the potential of using geotagged social media data to analyze population spatiotemporal activities, especially in emergencies.

## 1. Introduction

Along with the advances in communication and information technology and the popularity of smartphones and other mobile terminals, massive quantities of social media data are produced and propagated [1,2,3]. Social media sites such as Twitter, Facebook and Weibo provide venues for the public to share opinions, exchange insights and vent emotions without intermediaries or delay. On these sites, individuals, organizations and communities can share information in real time in the virtual world via their posts and sharing of content [4,5]. Therefore, researchers increasingly regard social media data as a new type of data to study human behavior at an unprecedented scale [6,7,8].

A mass gathering, which is a typical human behavior, is defined as an event attended by a large crowd of spectators and participants [9,10]. An emergency event at a mass gathering is a sudden, urgent, and usually unexpected event at a crowd gathering that can very easily become a crowd disaster. An example of this is the Shanghai New Year’s Eve stampede on 31 December 2014, when 36 people were killed and some 47 others were injured [4]. Similar to most other crowd disasters, one of the major reasons for this tragedy was that management officials incorrectly estimated the number and density of participants, and the impact of psychological factors, such as crowd mood, was not properly considered [4,11,12]. According to Berlonghi [13], the safety of a mass gathering event largely depends on crowd management, which is a challenging task because both human flow control and psychological monitoring must be simultaneously considered. Human stampedes, as a major cause of mortality in mass gatherings, are incompletely investigated complex phenomena, and prevention strategies remain insufficient [14,15].

A large quantity of social media data makes it possible to detect emergency events or the status of an assembled crowd. Leveraging social media big data for emergency response and disaster management has attracted considerable attention [16,17,18]. Social media data associated with temporal and geographical information can reveal human behavior patterns [19,20,21], and human emotion changes can be reflected by social media content [22,23,24,25]. Thus, many researchers have applied social media methods to study natural disasters such as earthquakes [26,27], forest fires [28,29] and floods [30,31]. Zheng et al. [32] aimed to detect urban emergency events such as fires, fights, and crashes based on social media data but did not discuss events such as crowd gatherings, which were treated as a process rather than an emergency. In addition, an increasing number of studies have explored ways to monitor crowds by predicting the daily routine trajectories of individuals. However, such an approach cannot detect crowd anomalies that will lead to crowd disasters in an emergency because crowd anomalies are generally caused by infrequent collective activities [11]. Zhou et al. [11] used big data from the Baidu map to establish early warnings of crowd disasters; however, they based the study mainly on users’ query data and did not consider users’ emotions. Ngo et al. proposed a crowd-monitoring framework [33] that combines an emotional analysis of Twitter data and widely-adopted crowd models. Martínez-Castaño et al. proposed a platform incorporating a huge amount of social media data for real-time analysis of the incidence of depression in the population. Nevertheless, these frameworks might be applicable only for English or Spanish posts, and they do not analyze the influence of spatiotemporal neighborhoods [34]. In general, most of the literature has focused on the effects of human activities on relatively extreme types of natural disasters or on daily routine behavior. Additionally, research on Chinese social media remains rather limited compared with studies of English-based services like Twitter and Facebook.

We employed social media big data to identify patterns, trends and associations relating to the 2014 Shanghai New Year’s Eve Stampede. Social media data-based approaches have advantages in accurately and multidimensionally tracking human mobility, reflecting crowd sentiment and identifying human behavior in an emergency. Techniques including spatial autocorrelation analysis, topic modeling and sentiment analysis in the Chinese context are applied in this research. To the best of our knowledge, the proposed work is the first to study crowd detection in mass gatherings based on social media data in Chinese, with spatiotemporal information. This work demonstrates the potential for further applications of data-driven emergency response and management.

This paper is structured as follows: Section 2 introduces the study area, experimental data and methods used in the research. Section 3 outlines the results of spatial autocorrelation, topic modeling and sentiment analysis. Section 4 summarizes contributions and suggests future work. Finally, Section 5 draws a conclusion.

## 2. Methodology

### 2.1. Study Case Description

**Figure 1:**
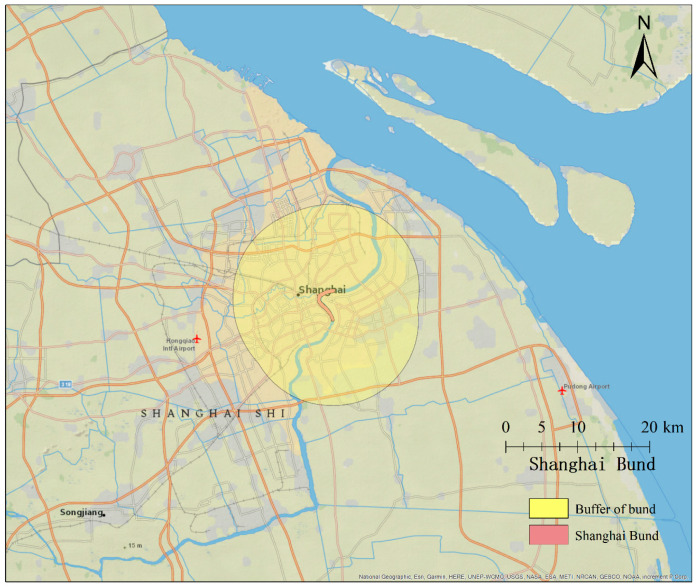
The Bund and corresponding buffer.

Shanghai, located in the Yangtze River Delta in the middle portion of the East China coast, had a population of more than 24 million in 2014. The administrative boundaries of the city are shown in Figure 1. The Shanghai New Year’s Eve stampede occurred near Chen Yi Plaza on the Bund on 31 December 2014. Approximately 310,000 people had gathered for the New Year celebration. The stampede was a disaster that killed 36 people and injured 49 others [4].

**Figure 2:**
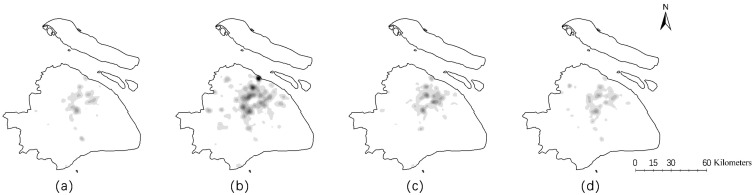
Check-in density between 12:00 on 31 December 2014 and 12:00 on 1 January 2015. Subfigures (**a**–**d**) show the spatial distribution of the check-in data during 31 December 2014 12:00–18:00, 31 December 2014 18:00–24:00, 1 January 2015 0:00–6:00 and 1 January 2015 6:00–12:00, respectively.

The Bund is a waterfront area in central Shanghai (shown in Figure 1). Centered on Zhongshan Road (East-1 Zhongshan Road), this area faces the modern skyscrapers of Lujiazui in the Pudong District. The Bund usually refers to the buildings and docks along this stretch of road, together with some adjacent areas. Chen Yi Plaza, lying in the middle of the Bund, links to the Huangpu River platform by a large and scenic staircase, which attracted a huge visitor flow, causing the highest population density during the New Year celebration [35]. In our research, we established a 10-km buffer zone for the Bund as representative of the Bund area (shown in Figure 2).

At approximately 20:00 on 31 December, more people were arriving at the Bund than were leaving. There was a continuous flow of visitors onto the platform, and they gradually huddled. According to the estimations from the government, there were approximately 120,000 people on the Bund at approximately 20:00, 160,000 from 21:00 to 22:00, 240,000 from 22:00 to 23:00 and 310,000 from 23:00 until the incident occurred. Finally, at 23:35, under pressure from the crowd, people at the bottom of the stairs were knocked to the ground, which triggered the eventual deadly stampede. As shown in Figure 2, the check-in density varied significantly over time, and the density from 18:00 to 24:00 on 31 December, when the stampede occurred, was apparently higher than at other times (shown in Figure 2b).

### 2.2. Sina Weibo Microblog Check-In Data

Sina Weibo is a popular social networking website in China (possessing 516 million active users as of April 2020) and has many similarities to the Twitter microblog. In addition, Weibo has been widely used in disasters and emergencies in China due to its sufficient data availability [36,37,38]. Individual users can post an emergency in near-real time, and the administrators utilize this information to broadcast warnings, notices and urgent news in a timely manner. We obtained the Weibo data from Weiboscope at the University of Hong Kong [39].

Check-in Weibos, accounting for approximately 1% of posts, are Weibo posts with geotags. Check-ins on social media involve users confirming their location and safety when an incident occurs at a specific location [40,41,42]. In addition, check-ins can involve users automatically sharing their location when they transmit messages on websites [43]. Sina Weibo check-in data include both types of location information. Thus, the data can provide geographic coordinate records of user activities and the context of user posts over a specific period.

During the Shanghai New Year’s Eve stampede, thousands of Weibos were posted. In this work, we collected all Weibos with a location in Shanghai, China, between 24 December 2014, and 7 January 2015 (one week before and after the Shanghai New Year’s Eve stampede). After filtering duplicate posts, 46,193 Weibos were used in the study.

### 2.3. Proposed Methods

**Figure 3:**
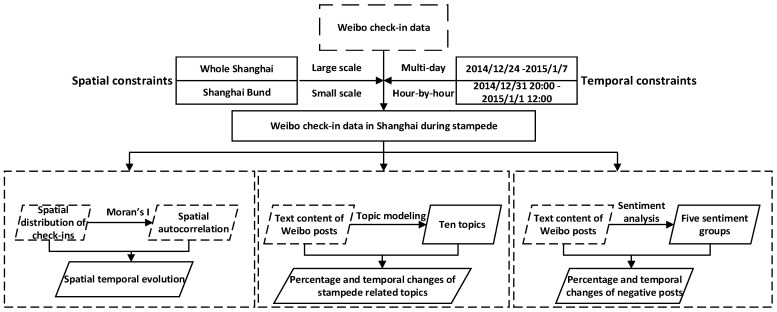
The framework for analyzing Weibo check-in data associated with the Shanghai New Year’s Eve stampede.

The analysis framework used in this paper is shown in Figure 3. The Weibo check-in data in this research were acquired from the data source by selecting the points within Shanghai between 24 December 2014, and 7 January 2015. We focus on the check-in data in Shanghai Bund for further investigation. The check-in data from 31 December 2014, at 20:00 to 1 January 2015, at 12:00 are analyzed hour-by-hour. The location information from Weibo check-in data over time are aggregated to analyze the temporal and spatial evolution of the stampede, mainly based on spatial autocorrelation analysis with Moran’s I. The text content of Weibo posts is analyzed by the topic modeling method, from which we derive ten stampede-related topics and identify the percentages of and temporal changes in them. Sentiment analysis is also applied to the text content of Weibo posts to categorize them into five groups, ultimately determining the percentages of and temporal changes in negative posts. In our work, this framework is applied to assess the practicality of social media data for crowd detection in mass gatherings.

#### 2.3.1. Spatial Autocorrelation

Spatial autocorrelation generally describes the correlation among variables considering the relative position dependence of spatial units [44]. If adjacent units tend to have similar values, a positive spatial autocorrelation appears, and if adjacent units tend to have different values, a negative spatial autocorrelation will appear [45]. The basic measure of spatial autocorrelation is the spatial autocorrelation coefficient. Moran’s I is the most common index, defined as the ratio between the local and global consistencies, as shown in Formula (1):

(1) $$I=\frac{n\sum_{i=1}^{n}\sum_{j=1}^{n}w_{ij}\left(x_{i}-\bar{x}\right)\left(x_{j}-\bar{x}\right)}{\sum_{i=1}^{n}\sum_{j=1}^{n}w_{ij}\sum_{i=1}^{n}{\left(x_{i}-\bar{x}\right)}^{2}}$$

where n denotes the number of spatial units, $x_{i}$ and $x_{j}$ denote the values of the ith and jth units, $\bar{x}$ denotes the mean of $x_{i}$, and $w_{ij}$ denotes the spatial weight matrix between the ith and jth units.

In our work, to quantify the distribution of check-ins, we establish regular grids to divide the study area and determine the quantity of check-ins in each grid. The grid size is $2^{\prime }\times 2^{\prime }$. We regard each grid as a basic spatial unit to calculate Moran’s index I.

#### 2.3.2. Topic Modeling and Sentiment Analysis in Chinese

Topic modeling aims at discovering hidden semantic information or knowledge in a large amount of text data. Latent Dirichlet allocation (LDA), as one of the most common topic models, is a generative probabilistic model and is specifically intended for usage on text corpora [46]. The model assumes that the document is a bag of words and analyzes the main discourse in the text corpus and divides the concurrent words into topics. Latent semantic analysis (LSA) is another important method to identify topics in a database of text. In this research, we select LDA instead of LSA because these two methods exhibit highly overlapping topics and LDA outperformed LSA in large test cases [47,48]. In addition, LDA has proved its adaptability for Chinese documents [49,50], while related work with LSA is limited.

In LDA models, each document can be regarded as a collection of latent topics, and each of the topics can be further illustrated by a set of keywords. Specifically, we use D to denote a text corpus, which is an assembly of documents, and $N_{D}$ to denote the number of documents in D; V is a set of words in documents, and $N_{V}$ is the number of words; $N_{T}$ is the number of topics. The generation process of the LDA model is as Algorithm 1 [51,52].
**Algorithm 1.** The Generation Process of the LDA Modelfor all topics $k\in \left[1,N_{T}\right]$choose a word distribution $\vec{\varphi_{k}}\sim \mathrm{dirichlet}\left(\vec{\beta }\right)$for all the documents $d\in \left[1,N_{D}\right]$choose a topic distribution $\vec{\theta_{d}}\sim \mathrm{dirichlet}\left(\vec{\alpha }\right)$for word $n\in \left[1,N_{d}\right]$choose the topic of the $n^{th}$ word:$\;Z_{d,n}\sim \mathrm{Multinomial}\left(\vec{\theta_{d}}\right)$choose the $n^{th}$ word: $W_{d,n}\sim \mathrm{Multinomial}\left(\vec{\varphi_{Z_{d,n}}}\right)$

In this procedure, $\vec{\varphi_{k}}$ is the word distribution for topic k; $\vec{\theta_{d}}$ is the topic distribution for document d. $\vec{\alpha }$ and $\vec{\beta }$ are the prior parameters for the topic-word distribution and the Dirichlet document-topic. $Z_{d,n}$ is the topic of the $n^{th}$ word in document d; $W_{d,n}$ is the $n^{th}$ word.

We select the open-source toolkits Jieba and Gensim for Chinese text segmentation and faster implementation of LDA, respectively. These toolkits are based on Hoffman [53], allowing both LDA model estimation from a training corpus and inference of topic distribution. As a result, the probability distribution of each Weibo post associated with the top 10 topics is computed and rescaled. In this work, we choose the topic that has the maximum probability as the Weibo topic.

Sentiment analysis is a natural language processing (NLP) task involving the computational treatment of opinions, sentiments and subjectivity in text [54,55,56]. In this research, we use the BosonNLP toolkit with an engine that uses the Weibo corpus for annotation and training. The BosonNLP toolkit provides a sentiment analysis application programming interface (API) especially for Weibo data.

## 3. Results and Discussion

### 3.1. Results of Spatial Autocorrelation

**Figure 4:**
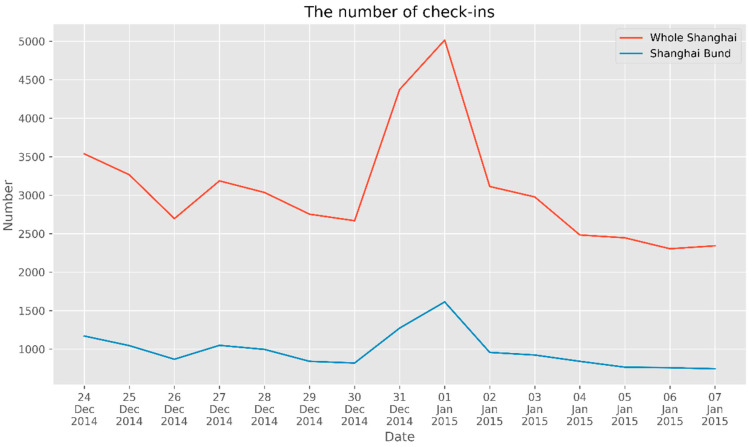
The number of check-ins in Shanghai and near the Bund.

**Figure 5:**
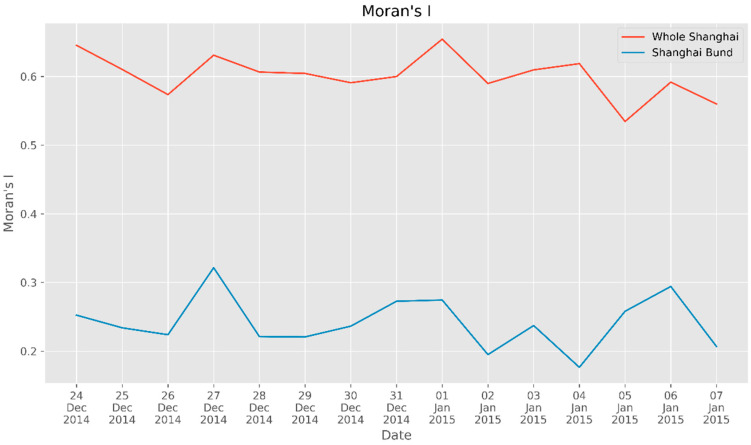
Moran’s I in Shanghai and near the Bund.

We calculated the number of check-ins in Shanghai and near the Bund each day from one week before to one week after the stampede (from 24 December 2014, to 7 January 2015), as shown in Figure 4. The red line in Figure 5 illustrates that the number of check-ins in Shanghai from 31 December 2014, to 1 January 2015 (the time of the disaster) was almost two times the peak value at other times. Additionally, the blue line, which represents the number of check-ins around the Bund, shows that there was a peak in Weibos during the disaster period. Furthermore, both the red line and the blue line show that the number of check-ins on New Year’s Eve in 2014 apparently exceeded the number of check-ins on Christmas Day.

Based on the method in Section 2.3, we calculate Moran’s I for the number of check-ins and plot the corresponding distribution over time. As shown in Figure 5, the red line is Moran’s I in Shanghai, with an average value of 0.601. The distribution of check-ins displays strong spatial autocorrelation with the municipal scale. In addition, we can observe several high-spatial aggregation areas in Shanghai. In particular, as indicated by the red line, the peak value of Moran’s I appeared on 1 January 2015. Combined with Figure 5, which shows a peak in the number of check-ins, we can conclude that the population density on New Year’s Eve was higher than at other times.

**Figure 6:**
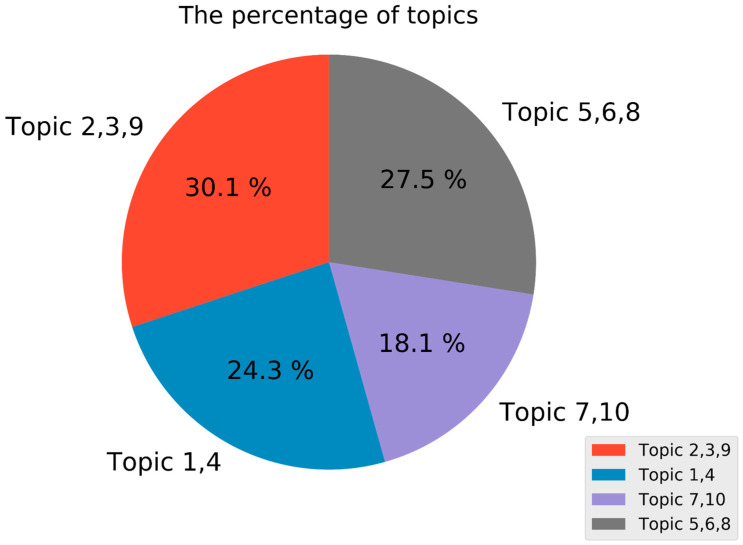
Contributions of ten topics to the percentage of each class.

The blue line in Figure 6 shows Moran’s I around the Bund, from which we can conclude that there was considerable spatial aggregation in the Bund area (the average value of Moran’s I was greater than 0), but this finding is not as obvious as that at the municipal scale. However, Moran’s I on New Year’s Eve was apparently higher than the index values on the days before and after the stampede, as well as the average value of the blue line.

In general, Moran’s I is higher for festivals and on weekends, suggesting that crowd spatial aggregation is more likely to occur on non-workdays. Combined with the crowd anomaly shown in Figure 4, we can intuitively detect the crowd flow and spatial aggregation distribution by analyzing the number and Moran’s I of check-ins.

### 3.2. Results of Topic Modeling

We extracted the posts made in the Bund area from 31 December 2014, at 18:00:00 to 1 January 2015, at 12:00:00 for topic modeling. In the preprocessing stage, we removed words related to the topic “happy New Year,” which were dominant at this special event and could potentially influence the topic modeling results. After preprocessing, 1280 posts were deemed valid and used to extract topics. To avoid a high degree of similarity between topics, while maintaining the clarity of each topic, we set the number of topics to 10. We defined a topic to be related to the stampede if at least one of the most frequent keywords was related to a crowd, stampede, or mass gathering. Seven of the 10 topics are related to the studied stampede, accounting for 72.4% of all posts. The topics are as follows.

Topic 1: There was a stampede in Shanghai Bund, I was fortunate that I missed it yesterday.
Topic 2: Hello, Shanghai Bund. There are many people walking here.
Topic 3: We are having fun among many people.
Topic 4: Good morning everybody. Wish you good luck. I feel blessed that I stayed with family yesterday.
Topic 5: I still feel happy despite the cold wave.
Topic 6: Happy days are coming with a very nice breakfast.
Topic 7: I will start working hard from the early morning.
Topic 8: Sharing songs for the dead. May the dead rest in peace.
Topic 9: Many people died tonight. We should stay calm in mass gatherings.
Topic 10: Hoping the dead from the stampede rest in peace.

The extracted 10 topics can be divided into four classes, and the percentage of each class is shown in Figure 6. The first class includes Topic 2, Topic 3 and Topic 9, which mainly express complaints about the huge crowds around the Bund. The key words for these topics are “huge crowd”, “Bund” and “death.” The posts associated with these topics account for 30.1% of all posts. The second class contains Topic 1 and Topic 4, and a common theme is people expressing relief about not going to the Bund. “Stampede”, “gloat” and “being alive” are words appearing frequently in these topics, and 24.3% of the posts are associated with such topics. The third class involves blessings and includes Topic 7 and Topic 10. The most important words for these topics are “rest in peace” and “pray for the deceased and injured.” Posts associated with these topics account for only 18.1% of all posts. The rest of the topics are not related to the stampede, including Topic 5, Topic 6, and Topic 8.

**Figure 7:**
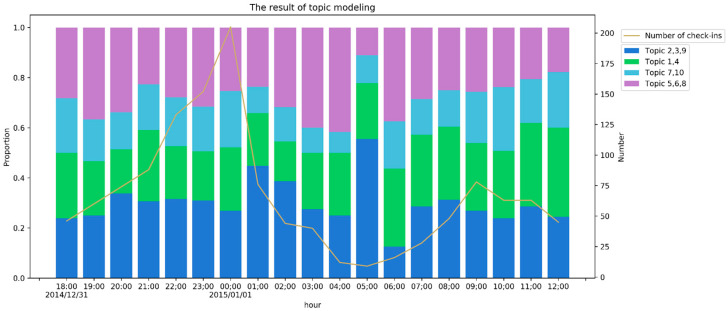
Usage frequency of each class from 31 December 2014 at 18:00:00 to 1 January 2015, at 12:00:00.

Figure 7 shows the usage frequency of each class in each hour from 31 December 2014 at 18:00:00 to 1 January 2015 at 12:00:00. The line chart in Figure 7 shows the number of check-ins in each hour. As shown in Figure 7, omitting three hours (1 January 2015 4:00–7:00) with very few check-ins, the proportion of the first class from 31 December at 20:00 to 1 January at 3:00 was 31.77%, higher than those at other times. The proportion of the first class from 1 January 2014, at 1:00 to 1 January 2015, at 2:00 was the highest, reaching 44.73%. Thus, people complained about crowding beginning at 20:00, which basically matches the process described in the report [4]. For the second class, the proportion of posts was higher after 1 January at 7:00 than on 31 December, suggesting that most people talked about the absence of celebration around the Bund the day after the event. For the check-in data outside the Bund in Shanghai, only one topic is related to the stampede. This low proportion also reflects the significant spatial autocorrelation characteristics of the stampede based on check-in data.

### 3.3. Results of Sentiment Analysis

In our research, we calculate a score between 0 and 1 for each post from 31 December 2014, at 18:00:00 to 1 January 2015, at 12:00:00 around the Bund. A high score indicates a positive post, and vice versa. For convenience in determining changes in sentiment, we divide the sentiment score into five groups with an interval of 0.2. Group 1 includes scores between 0 and 0.2, which is the group with the most negative sentiment; Group 5 includes scores between 0.8 and 1, which is the group with the most positive sentiment.

**Figure 8:**
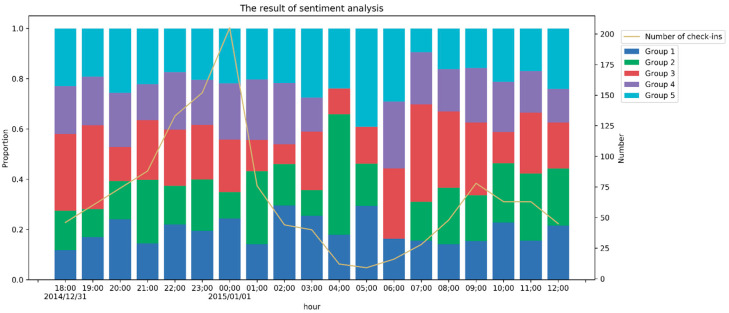
Usage frequency of each group from 31 December 2014 at 18:00:00 to 1 January 2015 at 12:00:00.

The histogram in Figure 8 shows the usage frequency of each group in each hour, and the line chart shows the number of check-ins in each hour. Excluding the three hours (1 January 2015 from 4:00–7:00) with very few check-ins, we can conclude that the proportion of negative sentiment (Group 1 and Group 2) increased in two periods: from 31 December at 20:00 to 1 January at 2:00 (40.95%) and on 1 January from 10:00 to 12:00 (44.33%). The first period was when the stampede occurred. In addition, Group 3 accounted for a minor proportion of posts during the event, suggesting that people shared an apparent sentiment inclination. For comparison, the proportion of negative sentiment (Group 1 and Group 2) from 31 December at 18:00 to 31 December at 20:00 is 27.83%.

## 4. Discussion

In this study, we propose an innovative framework from two dimensions of Weibo check-in data, geographic attributes and blog content, to explore the application of geotagged social media data in crowd detection. Social media data have outstanding advantages in tracking crowd change. This study demonstrates the potential of using geotagged social media data for analyzing population spatiotemporal activities, especially in emergencies.

(1) First, social media data are not limited by sparse sensor coverage. For example, in this study, only Weibo check-in data are used. Everyone in the stream is aware of the entire event timeline through the timestamp, geographic location, and semantic information in the social media data. Thus, processes such as event feedback do not require additional sensors or costs.
(2) Second, social media data are real-time data. The Weibo check-in data used in this paper can reveal the aggregation situation of the crowd in near-real time.

Social media data can be obtained in real time through the API provided by the social media operator. At the hourly and daily scales, social media data have proved effective for detecting anomalies in real time [57].

(3) Finally, social media data can be used in multidimensional analyses. For example, Weibo check-in data provide multiple features, such as space, time, and semantic features, to comprehensively analyze crowd change. Notably, the analysis of Weibo posts reflects the influence of user psychology and activities before and after the stampede. Performing such tasks is difficult based on video crowd detection [58] and mobile phone crowd detection [59]. Social media data can provide a new perspective for crowd gathering detection.

Based on these characteristics, social media data, especially geotagged social media data, can provide different dimensions for crowd detection and help the relevant government entities make good decisions.

However, the application of social media data in crowd detection still retains some challenges and requires further research. The main points are as follows.

(1) Improved data-filtering methods and more powerful NLP models are needed to improve the accuracy of the results.
(2) This research considers only Weibo posts and check-ins and does not include Weibo comments. In the future, we can add Weibo comment data and relationship chain information from comments to the analysis. For example, the impact range of the stampede event can be evaluated in combination with the comments. In cases of disaster recovery, this approach can enable more in-depth analysis of the event process.
(3) To efficiently extract relevant user information and classify microblog users, the current framework does not take user preferences into account. In future work, the features of different users can be extracted by using models, assigning different weights to Weibos or input features directly in an NLP model as parameters, and eliminating bias associated with user preferences in social media.

## 5. Conclusions

In this paper, we analyzed the crowd aggregation level, topic changes and sentiment changes of a crowd based on Weibo check-in data from the 2014 Shanghai New Year’s Eve stampede. For this purpose, we introduced Moran’s I in spatial autocorrelation analysis to quantitatively reflect the aggregation level and performed an analysis of posts in Chinese to reveal the psychological changes of users in the crowd. In addition, we established a 10-km buffer zone for the Bund and consequently differentiated the study area from the greater Shanghai area and the Bund area for analysis purposes. In addition, we divided the study area into equivalent grids to calculate Moran’s index I. We also considered changes in a given time period—from a week before to a week after the event—to examine people’s reactions in time and space.

The findings of this study are as follows. First, combined with the number of check-ins, Moran’s I can reveal the crowd aggregation level. The case study shows that a high index with an unusual number of check-ins suggests that a mass gathering has likely occurred. In this case, we can quantitatively detect the crowd flow and spatial aggregation distribution by calculating Moran’s I of check-ins. Second, the results of topic modeling show that the proportion of the crowd that complained about the huge crowds in the Bund area increased beginning 31 December at 20:00 and remained at a high level until 1 January at 3:00. Moreover, topics about the absence of celebration increased in proportion at 7:00 on 1 January. Finally, sentiment analysis based on the posts indicated that people with negative sentiments were most active from 31 December at 20:00 to 1 January at 2:00 (when the stampede occurred). In addition, the results demonstrate that, during the event, people generally showed an apparent sentimental inclination. We believe that the huge flows of people and the arrival of the new year triggered crowding, which led to both complaints and celebration.

The results show that it is feasible to detect crowd aggregation by social media data. On the one hand, the geographical attributes of social media can directly reflect changes in the flow of people over time; on the other hand, through analysis of Weibo posts, the effects of people’s psychological states before and after an incident can be identified. We believe that the proposed framework will be further applied in data-driven emergency response and management. Timely collected data via social media with geotags from cell phones was used to derive a real-time population distribution map and its spatiotemporally changing mode. Such massive accurate and multidimensional positioning data can be used to supervise the flow direction of crowds, control traffic, evacuate crowds and rescue wounded individuals. Additionally, using natural language-processing methods to identify people’s emotions in emergencies using social media data will aid in providing mental health care to help people overcome difficulties and recover from the disasters. Although general patterns can be reflected by social media data, there are still challenges in assessing the psychological status of a population with high precision. This is an open question in data-driven emergency response and management.

## References

[1] Young S.D. (2014). Behavioral insights on big data: Using social media for predicting biomedical outcomes. Trends Microbiol..

[2] Xiang Z., Gretzel U. (2010). Role of social media in online travel information search. Tour. Manag..

[3] Asur S., Huberman B.A. Predicting the future with social media. Proceedings of the IEEE/WIC/ACM International Conference on Web Intelligence and Intelligent Agent Technology.

[4] Brynielsson J., Johansson F., Jonsson C., Westling A. (2014). Emotion classification of social media posts for estimating people’s reactions to communicated alert messages during crises. Secur. Inform..

[5] He S., Zheng X., Zeng D., Luo C., Zhang Z. (2016). Exploring entrainment patterns of human emotion in social media. PLoS ONE.

[6] Ruths D., Pfeffer J. (2014). Social media for large studies of behavior. Science.

[7] Tyshchuk Y., Wallace W.A. (2018). Modeling human behavior on social media in response to significant events. IEEE Trans. Comput. Soc. Syst..

[8] Tyshchuk Y. (2015). Modeling Human Behavior in the Context of Social Media during Extreme Events Caused by Natural Hazards. PhD Thesis.

[9] Arbon P. (2004). The development of conceptual models for mass-gathering health. Prehosp. Disaster Med..

[10] Johansson A., Batty M., Hayashi K., Al Bar O., Marcozzi D., A Memish Z. (2012). Crowd and environmental management during mass gatherings. Lancet Infect. Dis..

[11] Zhou J., Pei H., Wu H. (2018). Early warning of human crowds based on query data from Baidu maps: Analysis based on Shanghai stampede. Big Data Support. of Urban. Planning and Management.

[12] Pretorius M., Gwynne S., Galea E.R. (2015). Large crowd modelling: An analysis of the Duisburg Love Parade disaster. Fire Mater..

[13] Berlonghi A.E. (1995). Understanding and planning for different spectator crowds. Saf. Sci..

[14] De Almeida M.M., Von Schreeb J. (2018). Human stampedes: An updated review of current literature. Prehosp. Disaster Med..

[15] Cheng Z., Lu J., Zhao Y. (2020). Pedestrian evacuation risk assessment of subway station under large-scale sport activity. Int. J. Environ. Res. Public Health.

[16] Song X., Zhang H., Akerkar R.A., Huang H., Guo S., Zhong L., Ji Y., Opdahl A.L., Purohit H., Skupin A. (2020). Big data and emergency management: Concepts, methodologies, and applications. IEEE Trans. Big Data.

[17] Xia T., Song X., Zhang H., Song X., Kanasugi H., Shibasaki R. (2019). Measuring spatio-temporal accessibility to emergency medical services through big GPS data. Health Place.

[18] Dai D., Wang R. (2020). Space-time surveillance of negative emotions after consecutive terrorist attacks in London. Int. J. Environ. Res. Public Health.

[19] Yin Z., Cao L., Han J., Luo J., Huang T. Diversified trajectory pattern ranking in geo-tagged social media. Proceedings of the 11th SIAM International Conference on Data Mining.

[20] Fujisaka T., Lee R., Sumiya K. Discovery of user behavior patterns from geo-tagged micro-blogs. Proceedings of the 4th International Conference on Uniquitous Information Management and Communication.

[21] Hasan S., Zhan X., Ukkusuri S.V. Understanding urban human activity and mobility patterns using large-scale location-based data from online social media. Proceedings of the 19th ACM SIGKDD International Workshop on Urban Computing.

[22] Ceron A., Curini L., Iacus S.M., Porro G. (2013). Every tweet counts? How sentiment analysis of social media can improve our knowledge of citizens’ political preferences with an application to Italy and France. New Media Soc..

[23] Yu Y., Duan W., Cao Q. (2013). The impact of social and conventional media on firm equity value: A sentiment analysis approach. Decis. Support Syst..

[24] Xia R., Jiang J., He H. (2017). Distantly supervised lifelong learning for large-scale social media sentiment analysis. IEEE Trans. Affect. Comput..

[25] Yang Y., Su Y. (2020). Public voice via social media: Role in cooperative governance during public health emergency. Int. J. Environ. Res. Public Health.

[26] Sakaki T., Okazaki M., Matsuo Y. (2012). Tweet analysis for real-time event detection and earthquake reporting system development. IEEE Trans. Knowl. Data Eng..

[27] Crooks A., Croitoru A., Stefanidis A., Radzikowski J. (2012). #Earthquake: Twitter as a distributed sensor system. Trans. GIS.

[28] De Longueville B., Smith R.S., Luraschi G. Omg, from here, I can see the flames!: A use case of mining location based social networks to acquire spatio-temporal data on forest fires. Proceedings of the 2009 International Workshop on Location Based Social Networks.

[29] Thomopoulos S.C.A., Kyriazanos D.M., Astyakopoulos A., Dimitros K., Margonis C., Thanos G.K., Skroumpelou K. OCULUS fire: A command and control system for fire management with crowd sourcing and social media interconnectivity. Proceedings of the SPIE Defense + Security.

[30] Cheong F., Cheong C. Social media data mining: A social network analysis of tweets during the 2010–2011 Australian floods. Proceedings of the Pacific Asia Conference on Information Systems (PACIS).

[31] Rosser J.F., Leibovici D., Jackson M.J. (2017). Rapid flood inundation mapping using social media, remote sensing and topographic data. Nat. Hazards.

[32] Xu Z., Zhang H., Liu Y., Mei L. Crowd sensing of urban emergency events based on social media big data. Proceedings of the IEEE 13th International Conference on Trust, Security and Privacy in Computing and Communications (TrustCom).

[33] Ngo M.Q., Haghighi P.D., Burstein F. A crowd monitoring framework using emotion analysis of social media for emergency management in mass gatherings. Proceedings of the 26th Australasian Conference on Information Systems.

[34] Martínez-Castaño R., Pichel J.C., Losada D.E. (2020). A big data platform for real time analysis of signs of depression in social media. Int. J. Environ. Res. Public Health.

[35] Zhou M., Wang M., Zhang J. (2017). How are risks generated, developed and amplified? Case study of the stampede incident at Shanghai Bund on 31 December 2014. Int. J. Disaster Risk Reduct..

[36] Shan S., Zhao F., Wei Y., Liu M. (2019). Disaster management 2.0: A real-time disaster damage assessment model based on mobile social media data—A case study of Weibo (Chinese Twitter). Saf. Sci..

[37] Xiao Y., Li B., Gong Z. (2018). Real-time identification of urban rainstorm waterlogging disasters based on Weibo big data. Nat. Hazards.

[38] Bai H., Yu G. (2016). A Weibo-based approach to disaster informatics: Incidents monitor in post-disaster situation via Weibo text negative sentiment analysis. Nat. Hazards.

[39] Fu K.-W., Chan C.-H., Chau M. (2013). Assessing censorship on microblogs in China: Discriminatory keyword analysis and the real-name registration policy. IEEE Internet Comput..

[40] Todd A.W., Campbell A.L., Meyer G.G., Horner R.H. (2008). The effects of a targeted intervention to reduce problem behaviors. J. Posit. Behav. Interv..

[41] Liu Y., Sui Z., Kang C., Gao Y. (2014). Uncovering patterns of inter-urban trip and spatial interaction from social media check-in data. PLoS ONE.

[42] Liu T., Yang L., Liu S., Ge S. (2017). Inferring and analysis of social networks using RFID check-in data in China. PLoS ONE.

[43] Zhen F., Cao Y., Qin X., Wang B. (2017). Delineation of an urban agglomeration boundary based on Sina Weibo microblog ‘check-in’ data: A case study of the Yangtze River Delta. Cities.

[44] Getis A. (2007). Reflections on spatial autocorrelation. Reg. Sci. Urban Econ..

[45] Schmal C., Myung J., Herzel H., Bordyugov G.V. (2017). Moran’s I quantifies spatio-temporal pattern formation in neural imaging data. Bioinformatics.

[46] Blei D.M., Ng A.Y., Jordan M.I. (2003). Latent Dirichlet allocation. J. Mach. Learn. Res..

[47] Cvitanic T., Lee B., Song H.I., Fu K., Rosen D. LDA v. LSA: A comparison of two computational text analysis tools for the functional categorization of patents. Proceedings of the 24th International Conference on Case-Based Reasoning.

[48] Williams T., Betak J. (2018). A comparison of LSA and LDA for the analysis of railroad accident text. Procedia Comput. Sci..

[49] Wu X., Fang L., Wang P., Yu N. Performance of using LDA for Chinese news text classification. Proceedings of the IEEE 28th Canadian Conference on Electrical and Computer Engineering (CCECE).

[50] Wang J., Peng Y., Wang Z., Yang C., Xu J. (2019). Topic mining of Chinese scientific literature research about “The belt and road initiative” based on LDA model from the Sub Disciplinary Perspective. Data Mining and Big Data, Proceedings of the 4th International Conference on Data Mining and Big Data, Chiang Mai, Thailand, 26–30 July 2019.

[51] Song Y., Pan S., Liu S., Zhou M.X., Qian W. Topic and keyword re-ranking for LDA-based topic modeling. Proceedings of the 18th ACM Conference on Information & Knowledge Management.

[52] Liu K., Gao S., Lu F. (2019). Identifying spatial interaction patterns of vehicle movements on urban road networks by topic modelling. Comput. Environ. Urban Syst..

[53] Hoffman M.D., Blei D.M., Bach F. Online learning for Latent Dirichlet Allocation. Proceedings of the 24th International Conference on Neural Information Processing Systems.

[54] Pang B., Lee L. (2008). Opinion mining and sentiment analysis. Found. Trends Inf. Retr..

[55] Nasukawa T., Yi J. Sentiment analysis: Capturing favorability using natural language processing. Proceedings of the 2nd International Conference on Knowledge Capture.

[56] Agarwal A., Xie B., Vovsha I., Rambow O., Passonneau R. Sentiment analysis of Twitter data. Proceedings of the Workshop on Languages in Social Media.

[57] Gu Y., Qian Z., Chen F. (2016). From Twitter to detector: Real-time traffic incident detection using social media data. Transp. Res. Part C Emerg. Technol..

[58] Xie S., Zhang X., Cai J. (2018). Video crowd detection and abnormal behavior model detection based on machine learning method. Neural Comput. Appl..

[59] Yuan Y. Crowd monitoring using mobile phones. Proceedings of the 6th International Conference on Intelligent Human-machine Systems & Cybernetics.

